# Reference ranges of light-adapted full-field electroretinogram and associated factors in a large cohort of healthy school-aged children and adolescents

**DOI:** 10.1007/s10633-025-10015-4

**Published:** 2025-03-22

**Authors:** Sonia Seen-hang Chan, Kai Yip Choi, Natalie Yu-yan Chan, Vivian Wai Ying Lo, Angela Hing-yiu Hung, Henry Ho-lung Chan

**Affiliations:** 1 Laboratory of Experimental Optometry (Neuroscience), School of Optometry, The Hong Kong Polytechnic University, 11 Yuk Choi Road, Hung Hom, Kowloon, Hong Kong SAR, China; 2 Centre for Myopia Research, School of Optometry, The Hong Kong Polytechnic University, Hong Kong SAR, China; 3 Centre for Eye and Vision Research (CEVR), 17W Hong Kong Science Park, Hong Kong SAR, China; 4 Research Centre for SHARP Vision (RCSV), The Hong Kong Polytechnic University, Hong Kong SAR, China

**Keywords:** Electroretinography, Refractive error, Axial length, Paediatric, Adolescents

## Abstract

**Purpose:**

This study aimed to establish a reference data set of light-adapted full-field electroretinograms (ffERG) of healthy primary and secondary schoolchildren and investigate the relationship among refraction, ocular biometry, and ffERG.

**Methods:**

Healthy children aged between 6 and 17 years were recruited. Cycloplegic spherical equivalent refraction (SER), axial length (AL) and keratometry (K) measurements were performed. Standardized ffERGs, including light-adapted flash and 30-Hz flicker, were measured using a portable device with skin electrodes. The percentiles of peak time and amplitude of a- and b-waves and 30-Hz flicker of ffERG waveform were reported, and their relationships with age, SER, AL, K were investigated.

**Results:**

Among the 445 participants (45.4% female), the SER (mean ± standard deviation) was − 0.72 ± 2.06 D and AL was 23.56 ± 1.15 mm. The 90% confidence interval (CI) of 5th reference limit of amplitudes of a- and b-wave were 5.0–5.8 µV and 15.0–17.6 µV, while 95th reference limit of peak times were 13.2–13.4 ms and 29.8–30.0 ms, respectively. The amplitude and peak time of the 30-Hz flicker waveform were 21.5–23.9 µV and 26.0–26.2 ms, respectively. In general, more myopic SER, and longer AL were associated with delayed and reduced ffERG waveforms. Older age and male sex were weakly correlated with ffERGs with minimal clinical significance.

**Conclusions:**

A reference data set of light-adapted ffERG in children and adolescents was established for clinical and research purposes.

**Supplementary Information:**

The online version contains supplementary material available at 10.1007/s10633-025-10015-4.

## Introduction

Electroretinogram (ERG) is an objective functional test to measure retinal electrical responses when a photopic stimulation is delivered to the retina. It is a valuable ophthalmic diagnostic tool, which has been adopted in research studies. Different protocols have been established for measuring the electrical activities of different retinal cells, of which the most frequently adopted ones are the standards established by the International Society for Clinical Electrophysiology of Vision (ISCEV) [1]. The full-field ERG (ffERG) utilises a homogenous flash to stimulate the whole visual field after mydriasis to standardise the amount of light entering the eye, and the overall response from the whole retina is collected [2, 3]. The protocol includes measurements of rod-mediated or rod-cone mixed retinal function of a dark-adapted eye in scotopic condition and cone-mediated retinal function of light-adapted eye in photopic condition [4]. The light-adapted ffERG is first measured with a standard flash at 2 Hz, in which the waveform includes a negative a-wave originating from photoreceptor and OFF-bipolar cells and a positive b-wave from Müller and cone ON- and OFF- bipolar cells that are mediated by L-, M- and S-cone mechanisms [2, 3]. Then a 30 Hz flickering stimulation is used. The responses to this stimulation originate from post-receptoral neurons representing the function of rapidly recovering cones [5]. Conventionally, an ERG test is indicated in children with various ocular circumstances, such as nystagmus, unexplained poor visual acuity, structural ocular abnormalities, and family history of inherited retinal diseases, to aid disease diagnosis and progression monitoring. The use of ERG has further expanded to paediatric research fields, including refractive error, colour vision, and myopia control safety investigations [6–8].

Myopia is the most common refractive error and its global prevalence is expected to reach 50% in 2050 [9]. It also been demonstrated there is geographical variation in prevalence and that Western countries have lower rates of myopia, while Eastern countries have a much high prevalence [10]. In particular, the prevalence of myopia in Hong Kong children aged 6–8 years was 25%, and childhood myopia prevalence in Hong Kong was the highest comparing to other regions around the world [11]. Both the surge in myopia prevalence and increase in severity in Hong Kong, with the increase in myopia prevalence from 18.3% at the age of 6 to 61.5% at the age of 12 years [12]. Myopia, especially high myopia, is associated with vision-threatening complications, including retinal detachment, myopic macular degeneration, and glaucoma [13]. Thus, it is important to control myopia early in childhood to prevent the progression to high myopia later in life.

Myopia is widely recognised as a retina-mediated condition, in that the retina responds to environmental stimuli and modulates eye growth locally [14]. Hence, correlations between refractive error, axial length and ERG waveforms have been previously reported. Adult subjects with higher myopic refraction or longer axial lengths were revealed to have lower scotopic and light-adapted a- and b-waves, and 30 Hz flicker amplitudes [15, 16]. A longitudinal study also reported reduced light-adapted a- and b-waves, and 30 Hz flicker waveforms in children aged 8–12 years [7] and reduced multifocal ERG responses in children aged 9–14 years [17]. Previous studies have also reported that the specific ERG protocol would help to predict future myopia progression in children [6, 18]. As myopia development involves retinal physiology, the relationships between refractive error, axial length and ERG waveforms have been investigated [19–21], but more studies in this area are essential due to relatively smaller sample sizes (around a hundred subjects) and limited conclusive results. In addition, paediatric ERG has faced challenges as it conventionally requires mydriasis, or even general anesthesia or sedation in infants and toddlers, leading to a lack of large-scaled paediatric ERG reference data sets. Previously, we have established a reference data set of light-adapted flash and flicker ERG, including data from 479 Chinese preschool children in Hong Kong aged 3–7 years, using the RETeval (LKC Technologies Inc., USA), a commercially available handheld portable ERG device [22]. It was optimized for paediatric measurements as it uses a skin electrode, which is better accepted by subjects than corneal electrodes directly in touch with the eye. The RETeval does not necessarily require mydriasis because of real-time pupillometric adjustments of light intensity and its ability to maintain a constant retinal illuminance. RETeval results were previously compared to those obtained using conventional ERG systems in both paediatric and adult patients, and revealed there was a high level of agreement [23]. The device is gaining popularity in various research fields because of its efficiency, portability, and convenience, such as diabetic retinopathy [24–26], glaucoma [27–29], and paediatric ophthalmology [30–32].

To further improve the generalization of paediatric ERGs, this study aimed to establish reference data of light-adapted ffERG in school-aged children and adolescents, as this population is facing increasing challenges to the retina including increasing reading time and screentime, starting of myopia control interventions, and increasing academic stress, which might affect circadian rhythm. This also supplements our previous reported data set on preschool children. Factors affecting ffERG were also investigated, including age, sex, refraction, and ocular biometry. The difference in the light-adapted ERG reference data of preschool and school-aged children, and the difference in demographics and trends between refractive error and ffERGs in the two populations are also discussed.

## Methods

This cross-sectional study was approved by The Hong Kong Polytechnic University Institutional Review Board (Reference number: HSEARS20220609001) and all procedures followed the tenets of the Declaration of Helsinki. Written assents and consents were collected from the subjects and their parents or guardians, respectively, before any study-related procedures.

### Subjects

Healthy subjects aged 6–17 years were recruited via street booths and advertisements in community parent groups. All subjects underwent visual acuity measurements and internal and external ocular examination to evaluate inclusion criteria: (1) best visual acuity of at least logMAR 0.00; (2) absence of binocular anomalies and amblyopia; (3) absence of any ocular diseases. Subjects with any known systemic diseases and history of myopia control interventions were also excluded. All measurements, including ERG, cycloplegic refraction, and biometry measurements were conducted in a single visit in the Optometry Research Clinic of The Hong Kong Polytechnic University.

### ERG measurement

Full-field ERG measurements were performed using a handheld portable ERG device, RETeval, with Sensor Strip skin electrodes. RETeval consists of a Ganzfeld dome, which stimulates the whole retina with a homogenous light stimulus. Sensor Strip skin electrodes are placed 2 mm below the lower eyelid of both eyes with nasal edge aligned to the pupil center at primary gaze. The ffERG measurement was performed after a light adaptation process in a well-illuminated room at around 300 lx. According to the International Society for Clinical Electrophysiology of Vision (ISCEV) standard, light-adapted flash and flicker ERG measurements without mydriasis is accepted with consistent retinal illumination with adequately compensated stimuli and background illumination [3]. RETeval consists of a built-in infra-red pupillometric camera which simultaneously monitors the subject’s fixation stability and adjusts the luminance to maintain constant retinal illuminance for real-time pupillometric changes.The built-in ISCEV troland protocol in RETeval was matched with the ISCEV standard candela protocol by assuming the pupil diameter as 6 mm (i.e., pupil area of 28.27mm^2^) with the following equations:

$${\text{Troland}} = \left( {{\text{luminance }}\;{\text{incd}}\;/{\text{m}}^{2} } \right) \times \left( {{\text{pupil}}\;{\text{ area}}\;{\text{ in}}\; {\text{mm}}^{2} } \right)$$

$${\text{Troland}} \cdot s = \left( {{\text{flash }}\;{\text{luminance }}\;{\text{incd}} \cdot s/{\text{m}}^{2} } \right) \times \left( {{\text{pupil }}\;{\text{area }}\;{\text{in mm}}^{2} } \right)$$

Thus, the RETeval troland protocol consists of 2-Hz and 28.3-Hz 85 Td·s white flash stimuli under 850 Td white background respectively, were performed without the use of any mydriatic agent. Subjects were instructed to focus on a central red-light target in the Ganzfeld dome throughout the whole measurement. Following the RETeval practical guidelines, the subjects kept both eyes open and uncovered to obtain good fixation in paediatric subjects. If the noise of the skin electrode is above 55 μV for 2-Hz flash test or 5600 μV for 28.3-Hz flicker test, the examiner would reposition the skin electrode to achieve a good signal-to-noise ratio before starting the examination. The whole ffERG examination process of both eyes, including preparation and data taking, lasted for around 5–10 min including breaks in between measurements.

### Refractive error and biometric measurements

Thirty minutes after instilling 2 drops of 1% cyclopentolate with a 5-min interval between instillations, cycloplegic objective refraction was performed using an open-field autorefractor (NVision K5001, Rexxam, Osaka, Japan). Five consecutive readings were measured, and the representative value of spherical equivalent refraction (SER) automatically generated by the autorefractor was recorded. Only readings within a variation of 0.50 D were accepted [33]. Ocular biometric measurements including axial length (AL) and keratometry (K) (the mean corneal curvature of steep and flat meridians) were measured by partial coherence interferometry (IOL Master 500, Carl Zeiss Meditec, Jena, Germany). The average value of 5 readings was obtained and reported.

### Statistical analysis

Data analysis was performed using the SPSS statistical package (version 28, IBM Inc., Armonk, NY, USA). The light-adapted ffERG, in terms of amplitudes and peak times of a-, b-waves and 30-Hz flicker waveforms, were reported as 90% confidence interval (CI) of one-sided lower 5th reference limit of amplitudes and upper 5th reference limit of peak times, and 5–95th percentiles. The CI was calculated by the following equation[34] where s is standard deviation and n is sample size:

$$90\% {\text{CI}} = {\text{reference}}\;{\text{ limit}} \pm 2.81\left( {\frac{s}{\sqrt n }} \right)$$

While 5–95th percentiles were calculated using ‘Haverage’ (using weighted average) method in SPSS software[35]. The relationships among ffERGs, age, SER, AL, and K were evaluated using Pearson’s test with a correlation coefficient r. FfERG in different sexes was compared and reported in t-score and Cohen's d value. In order to evaluate the contributions of age, AL and SER on the ERGs, a hierarchical multiple regression was conducted, with 3 blocks of variables. The first block included age as the predictor, with amplitude or peak time as the dependent variable. In the second block, AL was also included as the predictor variable, and in the last block, SER was also included as the predictor variable on top of age. Statistical significance was defined as *p* < 0.05. Refraction and biometry were taken in both eyes while ERG measurements were only taken in one randomly selected eye. Hence, data of refraction and biometry were analysed and presented on the eye with ERG measurements only.

## Results

### Demographic, refractive, and ocular biometric results

Of the total of 494 participants recruited, 445 were included in the data analysis, while others were excluded due to poor best-corrected visual acuity (N = 4), presence of amblyopia or other ocular diseases (N = 18), use of myopia control treatment (N = 15), and inability to complete all tests (N = 12). The subjects, of whom 202 (45.4%) were female, were aged 9.75 ± 2.83 years (mean ± standard deviation). SER was − 0.72 ± 2.06 D, ranging from − 7.88 D to + 9.00 D, AL was 23.56 ± 1.15 mm, ranging from 20.35 mm to 27.24 mm, and K was 7.72 ± 0.26 mm, ranging from 6.82 mm to 8.45 mm. Age was negatively correlated with SER (r = − 0.47, *p* < 0.001), positively correlated with AL (r = 0.51, *p* < 0.001), and independent of K (*p* = 0.72). Of these 445 subjects, 436 (98.0%) and 437 (98.2%) were able to complete 2-Hz flash and 30-Hz flicker ffERGs respectively, while the rest cannot complete either one of the measurements due to their intolerance to the light stimulation.

### Full-field ERG and its relationships with age and between sexes

**Fig. 1:**
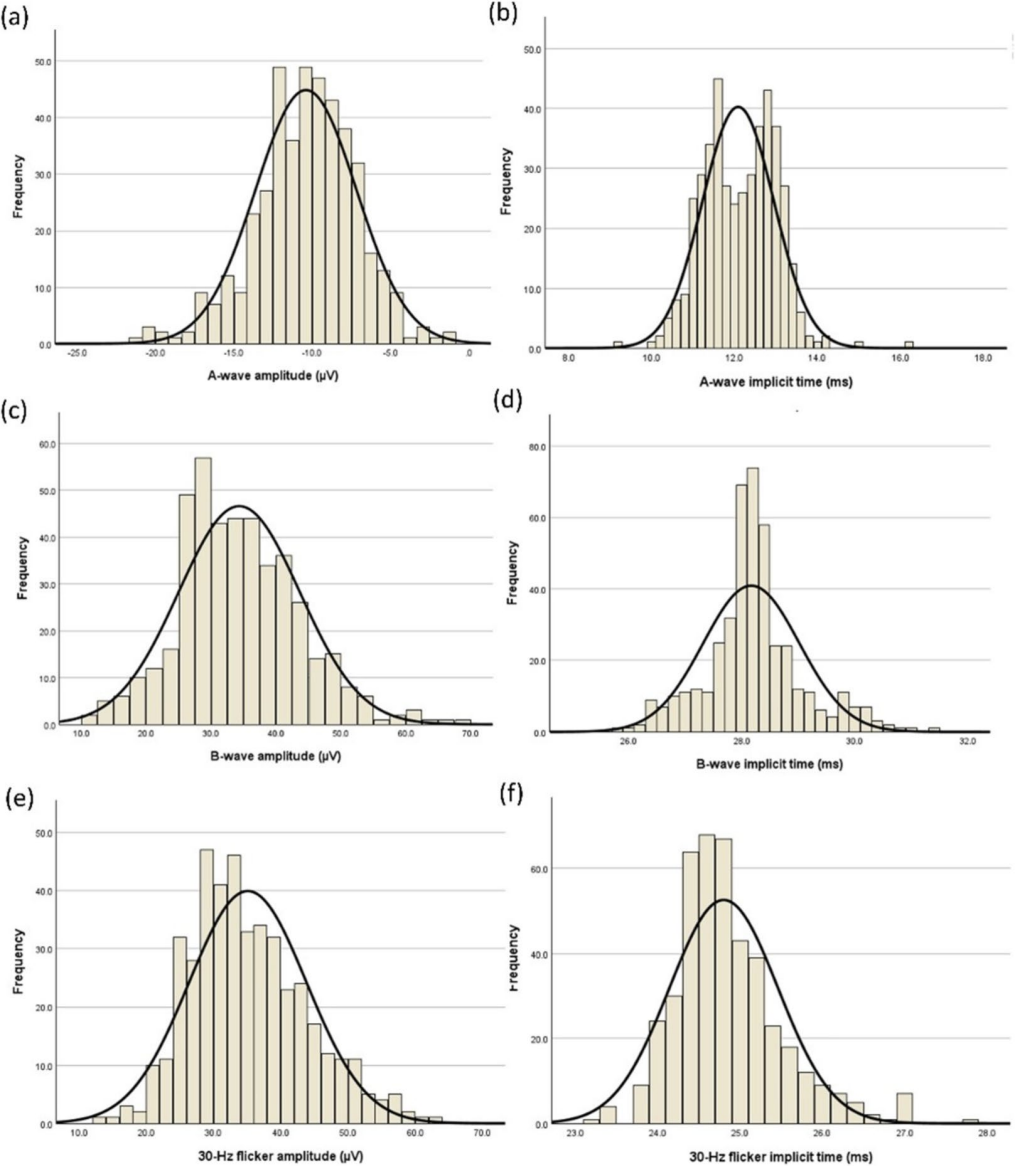
Distribution and Gaussian fit (solid line) of **a** a-wave amplitude **b** a-wave peak time **c** b-wave amplitude **d** b-wave peak time **e** 30-Hz flicker amplitude **f** 30-Hz flicker peak time

**Fig. 2:**
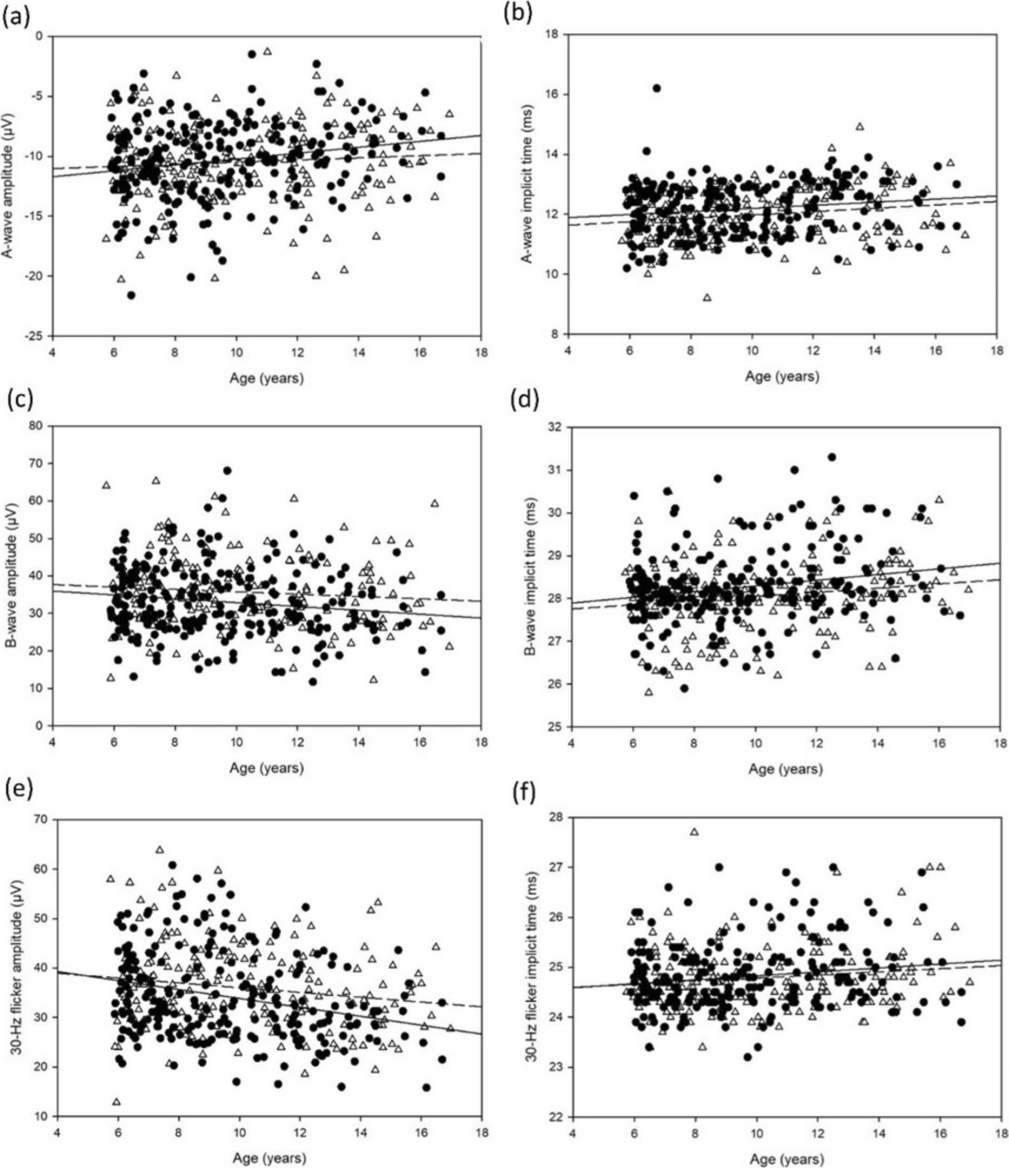
Pearson correlations between **a** a-wave amplitude **b** a-wave peak time **c** b-wave amplitude **d** b-wave peak time **e** 30-Hz flicker amplitude **f** 30-Hz flicker peak time and age. (Male: solid circle and solid line; Female: empty triangle and dotted line)

Figure 1 shows the distribution of the ffERG, and Table 1 shows the ffERG mean values and the 90% confidence interval (CI) of lower 5th reference limit of amplitudes and upper 5th reference limit of peak times of all subjects. The 5–95th percentiles are presented in Table 2. The a-wave amplitude (r = 0.146, *p* < 0.01), a-wave peak time (r = 0.167; *p* < 0.001), b-wave peak time (r = 0.181, *p* < 0.001), and 30-Hz flicker peak time (r = 0.119, *p* = 0.03) increased, while b-wave amplitude (r = − 0.114, *p* = 0.02) and 30-Hz flicker amplitude (r = − 0.218, *p* < 0.001) decreased with age. Although the effect sizes were small, males had longer a-wave peak times (t = − 2.61, *p* = 0.01, d = − 0.293), lower b-wave amplitude (t = 3.00, *p* < 0.01, d = 0.358), longer b-wave peak times (t = − 2.60, *p* = 0.01, d = − 0.341), and lower 30-Hz flicker amplitude (t = 2.14, *p* = 0.03, d = 0.283) than females, while no difference was found in a-wave amplitude (*p* = 0.67, d = − 0.119) and 30-Hz flicker peak times (*p* = 0.68, d = − 0.147). Figure 2 shows the relationships among age, sex, and ERG waveforms. The median difference between sexes of a-wave, b-wave and 30-Hz flicker amplitude were 0.2 uV, 2.7 uV and 3.1 uV, while those for peak times were 0.2 ms, 0.1 ms and 0 ms.

**Table 1:** 90% confidence intervals (CI) of the 5th percentiles (lower reference limits) of amplitudes and 95th percentiles (upper reference limits) of peak times of light-adapted a-waves, b-waves and 30 Hz flicker ERGs from all subjects

	Amplitude (uV) lower reference limit (90% CI)	Peak time (ms) upper reference limit (90% CI)
a-wave	5.4 (5.0–5.8)	13.3 (13.2–13.4)
b-wave	16.3 (15.0–17.6)	29.9 (29.8–30.0)
30-Hz flicker	22.7 (21.5–23.9)	26.1 (26.0–26.2)

*CI* confidence intervals

**Table 2:** The 5–95th percentile range of light-adapted a- and b-waves and 30-Hz flicker waveforms

All	Percentiles																		
	5th	10th	15th	20th	25th	30th	35th	40th	45th	50th	55th	60th	65th	70th	75th	80th	85th	90th	95th
Amplitude (μV)																			
A-wave	5.4	6.6	7.3	7.8	8.3	8.6	9.1	9.5	9.9	10.3	10.6	11.1	11.5	11.9	12.3	12.8	13.5	14.2	16.3
B-wave	16.3	23.7	26.0	26.8	27.8	28.9	29.7	31.3	32.4	33.7	34.9	36.1	37.5	38.6	40.2	42.1	43.5	46.3	50.2
30-Hz flicker	22.7	24.9	26.3	37.9	28.6	29.8	31.0	31.8	32.7	33.7	34.9	36.6	37.8	39.4	40.7	42.3	44.2	47.2	51.1
Peak time (ms)																			
A-wave	10.8	11.0	11.2	11.3	11.4	11.5	11.6	11.8	11.9	12.1	12.3	12.4	12.6	12.7	12.8	12.9	13.0	13.1	13.3
B-wave	26.7	27.1	27.5	27.6	27.8	27.9	28.0	28.0	28.1	28.1	28.2	28.2	28.3	28.4	28.5	28.7	28.9	29.2	29.9
30-Hz flicker	23.9	24.1	24.2	24.3	24.4	24.4	25.5	24.6	24.6	24.7	24.8	24.8	24.9	25.0	25.1	25.2	25.4	25.7	26.1

### Relationships among ffERG, SER, AL, and K

**Fig. 3:**
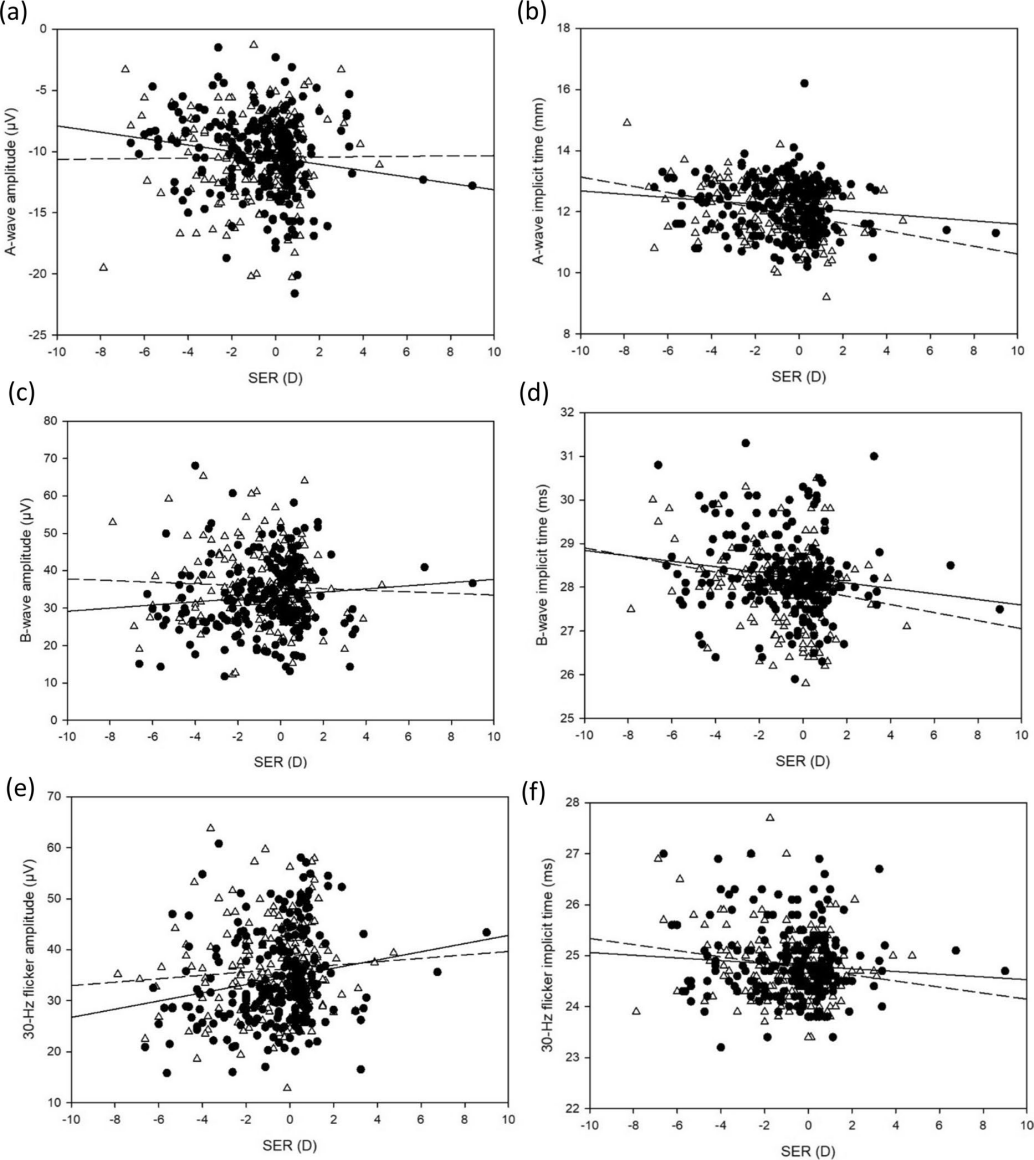
Pearson correlations between **a** a-wave amplitude **b** a-wave peak time **c** b-wave amplitude **d** b-wave peak time **e** 30-Hz flicker amplitude **f** 30-Hz flicker peak time and SER (Male: solid circle and solid line; Female: empty triangle and dotted line)

**Fig. 4:**
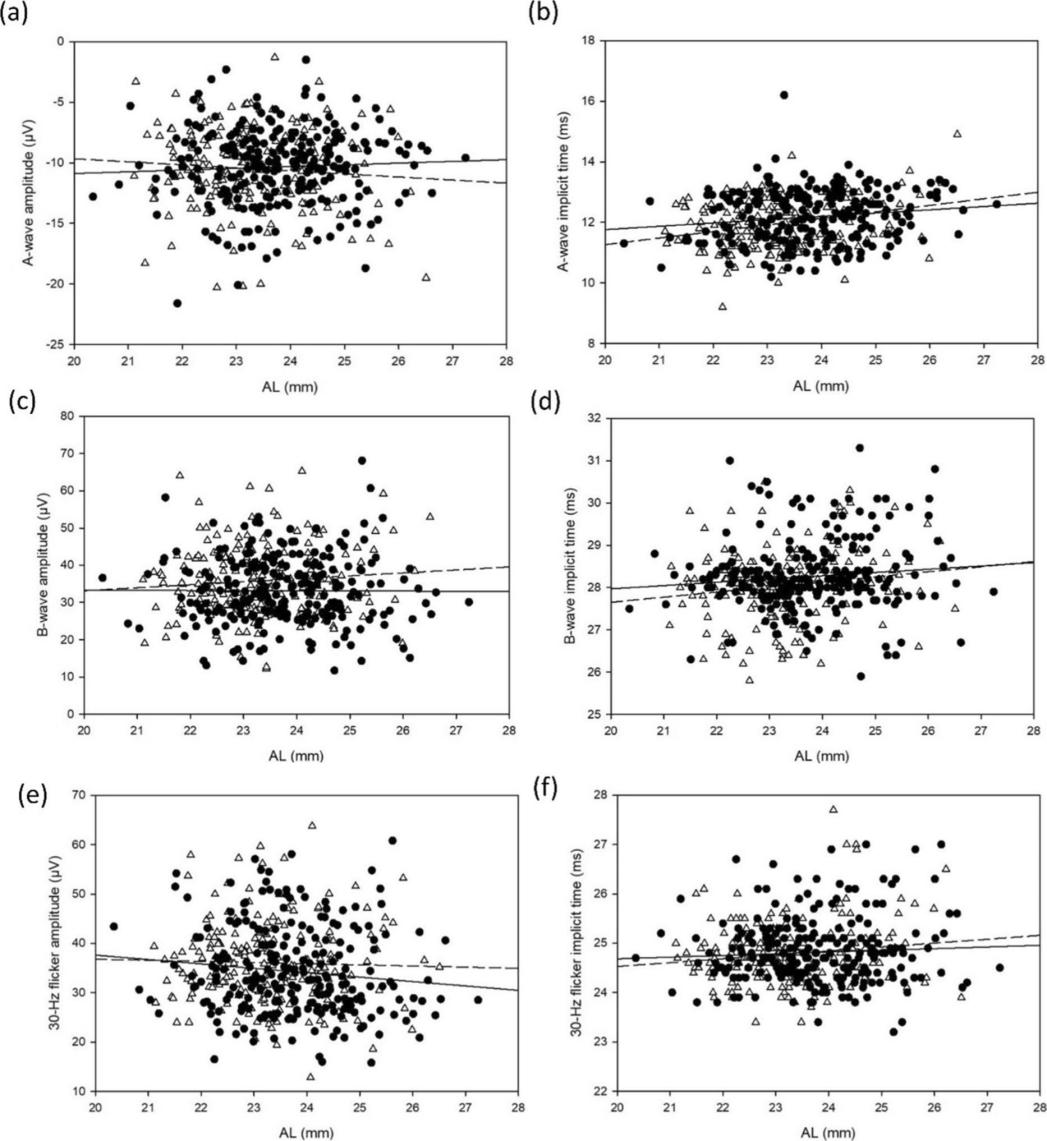
Pearson correlation between **a** a-wave amplitude **b** a-wave peak time **c** b-wave amplitude **d** b-wave peak time **e** 30-Hz flicker amplitude **f** 30-Hz flicker peak time and AL (Male: solid circle and solid line; Female: empty triangle and dotted line)

**Fig. 5:**
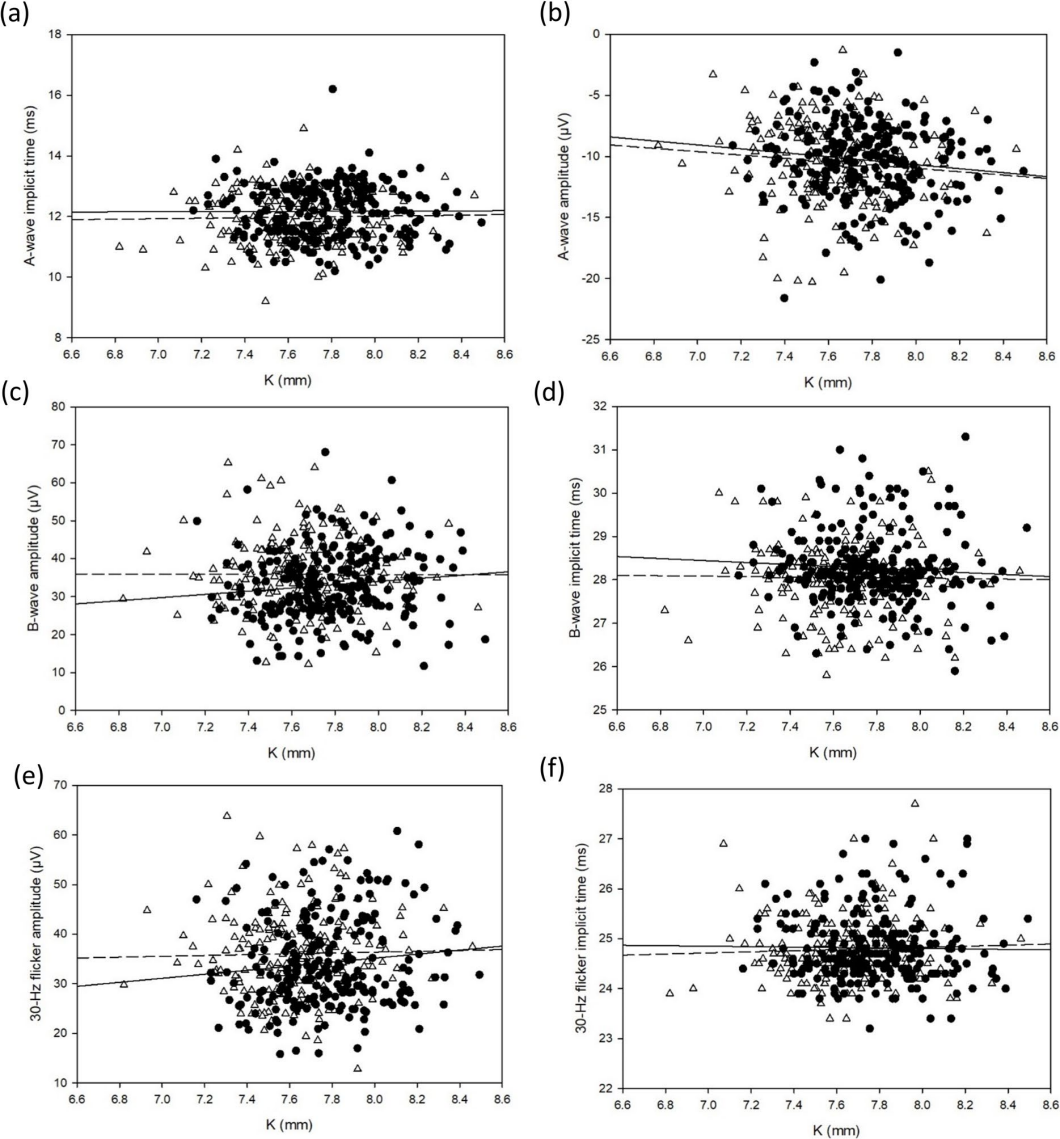
Pearson correlations between **a** a-wave amplitude **b** a-wave peak time **c** b-wave amplitude **d** b-wave peak time **e** 30-Hz flicker amplitude **f** 30-Hz flicker peak time and K (Male: solid circle and solid line; Female: empty triangle and dotted line)

A-wave peak time (r = − 0.197, *p* < 0.001), b-wave peak time (r = − 0.175, *p* < 0.001) and 30-Hz flicker peak time (r = − 0.126, *p* < 0.01) were negatively associated with SER, whilst 30-Hz flicker amplitude (r = 0.138, *p* < 0.01) was positively associated with SER. However, there was no statistically significant correlation found between a-wave amplitude (*p* = 0.06), or b-wave amplitude (*p* = 0.55). While AL was found to be positively correlated with a-wave peak time (r = 0.227, *p* < 0.001) and b-wave peak time (r = 0.169, *p* < 0.001), as well as weakly negatively correlated with 30-Hz flicker amplitude (r = − 0.100, *p* = 0.04), but not a-wave amplitude (*p* = 0.36), b-wave amplitude (*p* = 0.71), or 30-Hz flicker peak time (*p* = 0.05). No statistically significant correlation was found between K and ERG parameters except for a-wave amplitude (r = − 0.106; *p* = 0.03), which was negatively weakly correlated with K. Table 3 summarises the relationships among ffERG, refraction, and ocular biometry. Figures 3, 4 and 5 presents the relationships among ffERG, refraction, and ocular biometry.

**Table 3:** Correlations of a-wave and b-wave of light-adapted ERGs, 30-Hz flicker ERG waveforms with SER, AL and K in all subjects

Age (year)	A-wave		B-wave		30-Hz flicker	
	Amplitude	Peak time	Amplitude	Peak time	Amplitude	Peak time
SER	− 0.091 (*p* = 0.085)	− 0.197* (*p* < 0.001)	0.029 (*p* = 0.546)	− 0.175* (*p* < 0.001)	0.138* (*p* = 0.004)	− 0.126* (*p* = 0.008)
AL	− 0.002 (*p* = 0.966)	0.219* (*p* < 0.001)	0.007 (*p* = 0.877)	0.148* (*p* = 0.002)	− 0.100* (*p* = 0.037)	0.093 (*p* = 0.053)
K	− 0.106* (*p* = 0.028)	0.042 (*p* = 0.385)	0.020 (*p* = 0.671)	− 0.011 (*p* = 0.821)	0.044 (*p* = 0.355)	0.015 (*p* = 0.757)

An asterisk indicates statistical significance

### Multivariate analysis for the effect of age and AL, SER on ERG

Hierarchical regressions were adopted to evaluate the contributions of age, AL and SER on photopic flash and flicker ERG waveforms, while the statistical results of the hierarchical regressions were presented in Table 4. Age was found contributing to all photopic flash and flicker ERG parameters. For a-wave amplitude, on top of the 2.6% (*p* < 0.01) contribution by age, AL and SER contributed an additional 0.4% (*p* < 0.01) and 0.8% (*p* < 0.01) respectively. For a-wave peak time, on top of the 2.0% (*p* < 0.01) contribution by age, AL and SER contributed an additional 2.4% (*p* < 0.001) and 1.6% (*p* < 0.01) respectively. For b-wave amplitude, on top of the 1.5% (*p* = 0.02) contribution by age, AL contributed an additional 0.1% (*p* = 0.05) while the combined contribution of age and SER was statistically insignificant (*p* = 0.07). For b-wave peak time, on top of the 2.4% (p < 0.01) contribution by age, AL and SER contributed an additional 0.8% (*p* < 0.01) and 1.2% (*p* < 0.01) respectively. For 30-Hz flicker amplitude, on top of the 4.6% (*p* < 0.001) contribution by age, AL and SER contributed an additional 0.1% (*p* < 0.001) and 0.4% (*p* < 0.001) respectively. For 30-Hz flicker peak time, on top of the 1.4% (*p* = 0.03) contribution by age, SER contributed an additional 0.3% (*p* = 0.05) while the combined contribution of age and AL was statistically insignificant (*p* = 0.07).

**Table 4:** Hierarchical regression analysis of AL, SER, the combination of AL and age, and the combination of SER and age on light-adapted a-wave, b-wave, and 30-Hz flicker ERG waveforms

Models	Age			Age + AL			ΔR^2^	Age + SER			ΔR^2^	ΔR^2^
	R^2^	F	P value	R^2^	F	P value	(1 and 2)	R^2^	F	P value	(1 and 3)	(2 and 3)
Amplitude (μV)												
A-wave	0.026	9.48	0.002*	0.030	5.49	0.005*	0.004	0.027	4.93	0.008*	0.001	-0.003
B-wave	0.015	5.50	0.020*	0.017	3.03	0.050*	0.002	0.015	2.74	0.066	0.000	-0.002
30-Hz flicker	0.046	17.29	< 0.001*	0.047	8.72	< 0.001*	0.001	0.050	9.35	< 0.001*	0.004	0.003
Peak time (ms)												
A-wave	0.020	7.15	0.008*	0.044	8.12	< 0.001*	0.024	0.036	6.60	0.002*	0.016	-0.008
B-wave	0.024	8.83	0.003*	0.032	5.90	0.003*	0.008	0.036	6.58	0.002*	0.012	0.004
30-Hz flicker	0.014	5.07	0.025*	0.015	2.75	0.066	0.001	0.017	3.03	0.050*	0.003	0.002

Asterisk indicates statistical significance

## Discussion

The use of ERG is now gaining popularity in clinical practice and research studies owing to the convenience of measurements, particularly for paediatric ophthalmology. In this study, a reference light-adapted ffERG data set on school-aged children and adolescents aged 6–17 was established, which was a continuation of our recent study on preschool children aged 3–7 years in Hong Kong. Whether the clinical significance and expectations can be fulfilled by a reference dataset, can be reflected by the 90% CI, addressing the reliability of reference limits. In the current study, the 90% CI of the reference limits fell within 0.2 of the whole reference interval, indicating a good reliability of the current dataset [34]. All light-adapted ffERGs were found to be weakly age-dependent and with minimal clinical significance. More myopic SER was associated with longer a- and b-wave, and 30-Hz flicker peak times, while longer AL was associated with longer a- and b-wave peak times.

### Paediatric and adolescent reference data

Several other studies have collected ffERG data from healthy paediatric and young adult patients, but their sample sizes were smaller, and the age ranges wider than the current study. Soekamto and co-workers performed light-adapted flash and flicker ERG in 20 healthy subjects aged 4–17 years [36]. Nakamura and co-workers measured 30-Hz flicker ERG in 50 healthy subjects aged 4–56 years with only 10 subjects aged 4–17 years [37]. Liu and co-workers measured ERG using an ISCEV 5-step protocol in 57 healthy subjects aged 8–65 years with 25 subjects aged 8–19 years [38]. A recent study by Wang and co-workers also measured light-adapted flash ERG in 214 healthy subjects aged 6–12 with Ag–AgCl skin electrodes instead of RETeval sensor strips adopted in the other previous studies [39]. Relatively lower amplitudes and longer peak times were reported in their study, but these differences may be attributable to the types of skin electrodes used in the studies. Zhang and colleagues collected RETeval flicker ERGs from 204 healthy subjects aged 0–18 years in China and the United States, and presented locally-weighted-scatterplot-smoothing regression best fit equations and curves of flicker ERG amplitude and peak times [40], which estimated the ffERG parameters of different ages. Greater flicker amplitude but similar peak times were also observed in this study when comparing their age-matched results retrieved from the regression best fit curves. As the current study employed a larger sample size and a more focused age range, the data obtained should be more representative as a reference for clinical and research studies in paediatric and adolescent retinal physiology using the portable RETeval ERG device.

### Relationship between ERG, age and sex

The current study, by univariate analysis, revealed smaller a-wave amplitude, and longer a-wave, b-wave, and 30-Hz flicker peak times, but decreasing b-wave and 30-Hz flicker amplitude with increasing age. However, hierarchical regressions showed that age only contributed a small fraction of the variance and the small variation in amplitude or peak time might likely be the inter-session variability of similar ERGs [41]. Thus, the clinical contribution of age dependence in ERG waveforms might be small. Whilst the previous study performed on Hong Kong Chinese preschool children revealed increasing amplitudes and delayed peak time in b-wave and 30-Hz flicker ERGs with age. Soekamto et al. [36], performing flash and flicker light-adapted ERG in children aged 4–17 years, showed a strong positive correlation between age and a-wave peak time, which was similar to the results obtained in the current study. Wang et al. also reported slightly longer b-wave peak times in subjects aged 6–8 years than those in aged 9–12 years, but no differences were found in b-wave amplitude, a-wave amplitude and a-wave peak time[39]. However, Grace et al. found no correlation between 30-Hz flicker ERGs and age [42], and the exponential age-dependence best-fit curve reported by Zhang and colleagues showed a decrease in peak time before age of 11, after which a plateau was observed [40]. We speculate the difference in results obtained in preschool and school-aged children may indicate the physiological changes in the outer retinal layer as the children grow.

Zhang also reported no statistically significant difference in 30-Hz flicker ERGs between males and females [40], but lower 30-Hz flicker amplitudes in males were observed in the current study, which may be associated with longer axial length in males than females. Kato et al. [43] also reported that females had greater flicker ERG amplitudes when compared to males at the same age while Grace et al. noted that females had larger amplitudes and shorter peak times than males [42]. However, analysis of ffERGs in Singapore children revealed no statistical difference between males and females [7]. The current study also revealed that younger male subjects had stronger a-wave amplitudes than female subjects, while older male subjects had weaker a-wave amplitudes than females. However, the Cohen’s d values were low, showing the effect size of sex was small. Thus, the median difference between sexes of amplitudes and peak times may not be clinically significant.

### Relationship between ERG, SER and AL

The peak time of a-, b-wave and 30-Hz flicker increased, and 30-Hz flicker amplitude was slightly smaller in subjects with more myopic SER, while longer AL was found to be significantly correlated with longer a- and b-wave peak time and slightly correlated with lower 30-Hz flicker amplitude under univariate analysis, but the significant correlations were weak which contributed to a limited clinical significance. Multivariate analysis also revealed statistically significant but weak contributions from AL and SER on ERGs on top of the contribution by age. On top of the contribution by age (2%) on a-wave peak time, AL (2.4%) had a greater additional contribution than SER (1.6%). SER (1.2%) also made an additional contribution on b-wave peak time on top of the contribution by age (2.4%). While AL and SER only had weak additional contributions (less than 1%) or statistically insignificant contributions on a-wave amplitude, b-wave amplitude, 30-Hz flicker amplitude and peak time on top of the contribution by age. Results from the earlier study of Hong Kong Chinese preschool children showed that longer axial length was associated with smaller a-wave amplitude, greater b-wave and 30-Hz flicker amplitudes, longer a-wave peak time and shorter 30-Hz flicker peak time [17]. However, the peak times of a-wave and b-wave were found to be negatively associated with SER in school-aged children, but not in preschool children. Sachidanandam et al. [21] reported a decrease in amplitude and a delay in peak time with increasing AL, ranging from 21.79 to 30.55 mm, in young adults with mean age of 22.01 ± 5.6 years. However, Wang et al. found no such correlations between light-adapted flash ERGs with AL [39]. Westall et al. [20] showed a significant decrease in ERG amplitudes with increasing AL, ranging from 22.2 to 30.0 mm, in subjects aged 13–37 years. Similarly, Kato et al. [43] reported that longer AL was an independent factor associated with longer peak time of 30-Hz flicker ERG in young adults aged 20–29 years. This correlation was not found in the current study, but this discrepancy may be due to the different AL range and age range.

Graphical presentations in the current study also illustrated different trends of amplitudes and peak times against SER in between the sexes of the subjects. Male subjects with greater myopic refractive error had smaller amplitudes and shorter peak times than females, while male subjects with more hyperopic refractive error had stronger amplitudes and longer peak times than females. This suggests that sex difference in ffERGs against refractive error was present, but due to the small effect size, the clinical significance of sex on ERG waveforms was small.

### Limitations

Young subjects may not tolerate the bright stimulus, resulting in excess eye movements, leading to artifacts and, thereby, possibly lowering the reliability of the results. However, the built-in pupil tracking camera aiding the examiner in monitoring both the eye movements and fixation stability of the subjects, the auto-reject function removing faulty measurements, and the short measurement duration preventing fatigue and poor concentration may have ameliorated the limitation. Another possible limitation was that the mydriatic agent was not applied as in conventional measurements, although the real-time pupillography can adjust the stimulus intensity during measurement to prevent results being affected by non-dilated pupils [44]. Moreover, even with a fixed troland corrected with real-time and pupil size, the true retinal illuminance received by an eye with shorter axial length is greater than that in an eye with longer axial length [45], thus leading to a greater amplitude recorded in ERG measurements in the shorter eyeball. In the current study, the relationships between AL and ERG waveforms were minimal. Whether this AL-ERG independence was due to true retinal illuminance warrants further studies.

In conclusion, this study established a set of reference data of light-adapted ffERG for school-aged children using a portable ERG device for clinical applications and research studies. Correlations between light-adapted ffERG waveform characteristics and age, sex, SER, AL were revealed suggesting ongoing changes in retinal functions in children during their developmental years.

## Supplementary Information

Below is the link to the electronic supplementary material.

Supplementary file1 (DOCX 39 kb)

## Declarations

### Statement on the welfare of animals

No animals were used in this study.
