# Supplementary material for: Reference ranges of light-adapted full-field electroretinogram and associated factors in a large cohort of healthy school-aged children and adolescents
Source: Doc Ophthalmol. 2025 Mar 22;151(1):3–17. doi: 10.1007/s10633-025-10015-4 (PMC12334543; doi:10.1007/s10633-025-10015-4)
Supplement: Supplementary file 1 — Supplementary file1 (DOCX 39 kb) [file 10633_2025_10015_MOESM1_ESM.docx]

**SUPPLEMENTARY MATERIAL**

**Title:** Reference ranges of light-adapted full-field electroretinogram and associated factors in a large cohort of healthy school-aged children and adolescents

**Authors:** Sonia Seen-hang Chan^1,2^, Kai Yip Choi^1,2^, Natalie Yu-yan Chan^1,2^, Vivian Wai Ying Lo^1^, Angela Hing-yiu Hung^1,2^, Henry Ho-lung Chan^1,2,3,4^

**Institutions:**

^1^Laboratory of Experimental Optometry (Neuroscience), School of Optometry, The Hong Kong Polytechnic University, Hong Kong SAR, China

^2^Centre for Myopia Research, School of Optometry, The Hong Kong Polytechnic University, Hong Kong SAR, China

^3^Centre for Eye and Vision Research (CEVR), 17W Hong Kong Science Park, Hong Kong SAR, China

^4^Research Centre for SHARP Vision (RCSV), The Hong Kong Polytechnic University, Hong Kong SAR, China

**Correspondence to:**

Henry HL Chan

School of Optometry, The Hong Kong Polytechnic University, 11 Yuk Choi Road, Hung Hom, Kowloon, Hong Kong SAR, China

Phone: (852) 2766 7937

Email: [henryhl.chan@polyu.edu.hk](mailto:henryhl.chan@polyu.edu.hk)

**[Supplementary Table 1]** Correlation coefficients of a-wave and b-wave of light-adapted ERG responses, 30-Hz flicker responses with age

|  | Age | |
| --- | --- | --- |
|  | Correlation coefficient (r) | P value |
| *Amplitude (μV)* | | |
| A-wave | 0.146 | 0.002 |
| B-wave | -0.114* | 0.017 |
| 30-Hz Flicker | -0.218* | <0.001 |
| *Peak time (ms)* | | |
| A-wave | 0.176* | <0.001 |
| B-wave | 0.181* | <0.001 |
| 30-Hz Flicker | 0.150* | 0.002 |

*An asterisk indicates statistical significance*
